# Sequential UV-C Irradiation and *Sphingopyxis* sp. m6 Biodegradation for Enhanced Degradation and Detoxification of Microcystin-LR

**DOI:** 10.3390/toxins18030136

**Published:** 2026-03-10

**Authors:** Qin Ding, Tongtong Liu, Zhuoxiao Li, Rongli Sun, Juan Zhang, Lihong Yin, Yuepu Pu

**Affiliations:** Key Laboratory of Environmental Medicine Engineering, Ministry of Education, School of Public Health, Southeast University, Nanjing 210009, China; dingqin@seu.edu.cn (Q.D.);

**Keywords:** microcystin, UV-C, *Sphingopyxis* sp., sequential degradation, water treatment technology

## Abstract

Microcystins (MCs), a group of potent hepatotoxins from cyanobacterial blooms, threaten global water security due to the resistance to conventional treatment processes and multi-organ toxicity to human. This study innovatively proposed a novel sequential process combining UV irradiation with biodegradation by *Sphingopyxis* sp. m6 for efficient microcystin-LR (MC-LR) removal. Results revealed that sequential UV-C pretreatment followed by *Sphingopyxis* sp. m6 biodegradation achieved complete degradation of 1 mg/L of MC-LR within 1 h of the biological phase, drastically reducing the treatment time compared to biodegradation alone (5 h). Mechanistic investigation revealed that low-dose UV-C (50 mJ/cm^2^) pretreatment induced MC-LR photoisomerization consistently with previously reported Adda geometric isomers. These photoisomers, along with residual parent MC-LR, were subsequently mineralized by *Sphingopyxis* sp. m6. Enzymatic pathway analysis confirmed a dual-pathway degradation, where Mlr enzymes processed both the native toxin and its isomeric forms, leading to a series of linearized peptides and Adda-derived products. Critically, the process achieved efficient detoxification, as confirmed by the restoration of HepG2 cell proliferation and protein phosphatase 2A activity. Moreover, response surface methodology optimized the key parameters (31.49 °C, pH of 7.36, 0.23 mg/L) for the highest degradation efficiency. This work provides an energy- and cost-efficient strategy for MC-LR remediation and elucidates the molecular mechanism of UV-induced photoisomerization facilitating subsequent biodegradation.

## 1. Introduction

The proliferation of harmful cyanobacterial blooms, intensified by global eutrophication and climate change, has led to the widespread release of microcystins (MCs) into freshwater ecosystems, posing a serious threat to drinking water safety and public health [1,2,3,4,5,6]. Among over 300 variants, microcystin-LR (MC-LR) is one of the most prevalent and toxic congeners, primarily known for its potent inhibition of protein phosphatases 1 and 2A (PP1 and PP2A), which can lead to hepatocyte apoptosis and is implicated in tumor promotion [7,8]. Globally, most of freshwater is at risk of cyanobacterial blooms, during which MC-LR concentrations can reach dozens of folds higher than the WHO drinking water guideline (1 μg/L) [9,10,11]. Conventional water treatment processes, such as coagulation and filtration, are largely ineffective against dissolved toxins, while chemical disinfection like chlorination may generate toxic by-products [12,13]. Consequently, developing efficient, safe, and reliable methods for MC removal is an urgent and persistent challenge to public health.

Current primary degradation strategies include physical adsorption, advanced oxidation processes (AOPs), and biodegradation, yet each presents significant limitations [14,15,16]. Physical adsorption using materials like activated carbon is operationally simple but suffers from finite capacity, competitive adsorption in complex matrices, and the challenge of saturated adsorbent regeneration, essentially transferring rather than destroying the toxins [17]. AOPs, such as UV/H_2_O_2_ or ozonation, can rapidly degrade MC-LR, but their high energy and chemical reagent requirements, coupled with the potential generation of incompletely detoxified transformation products, limit their sustainability and application scope [18,19]. In contrast, biodegradation offers a promising route for complete mineralization and detoxification, utilizing native bacterial enzymes like the Mlr complex to cleave MC-LR into non-toxic, assimilable small molecules [20,21,22]. However, its practical application is constrained by the typically slow degradation kinetics, pronounced lag phases, and the sensitivity of microbial activity to fluctuating environmental conditions such as temperature and pH [23,24,25].

Single-treatment technologies often exhibit pronounced strengths and weaknesses in pollutant remediation. Integrating two or more processes offers a promising strategy to achieve enhancement of degradation efficiency and detoxification through complementary advantages [14,25]. Most existing studies focus on coupling physicochemical techniques, such as those coupling visible light with nanocomposites, UV with TiO_2_ or H_2_O_2_, or activated carbon with oxidation processes [18,26,27,28,29,30]. While physicochemical methods are typically efficient and rapid, they often suffer from high costs, chemical reagent requirements, incomplete detoxification, and toxic by-product formation. These drawbacks contrast with the safety, cost-effectiveness, and environmental compatibility of biodegradation, suggesting strong potential for complementary integration [14]. While integrating complementary technologies is a recognized strategy to overcome individual limitations, research on coupling physicochemical methods with biodegradation for MC remediation remains limited.

Ultraviolet (UV) can degrade various algae and cyanotoxins rapidly, generating macromolecular intermediates, but often fails to achieve complete mineralization and detoxification [31,32,33]. On the other hand, biodegradation (e.g., through bacterial community, *Sphingopyxis* sp.) can completely mineralize and detoxify MCs but suffer from lag phases and relatively slow degradation rate [20,21]. While combining UV and biodegradation methods was validated for degrading relevant compounds (sulfadiazine and pyridine), no studies have specifically evaluated UV irradiation–biodegradation coupling for MC-LR degradation [34,35]. This coupling approach may provide a low-cost, efficient, and safe strategy for MC degradation. Previous studies on UV-based MC-LR degradation have primarily focused on direct photolysis mechanisms and advanced oxidation processes (AOPs). Tsuji et al. [36] first reported that UV irradiation induces geometric isomerization of the Adda moiety in MC-LR, generating products such as 4(Z)-Adda MC-LR and 6(Z)-Adda MC-LR. Subsequent studies by He et al. [37] and Chintalapati and Mohseni [33] systematically investigated UV-C (254 nm) photolysis kinetics and demonstrated that while UV-C efficiently degrades MC-LR, complete mineralization is not achieved. Chang et al. [26] explored UV combined with ozone for MC-LR oxidation, identifying various degradation products, but the biological fate and toxicity of these UV-generated intermediates remained unexplored. More recently, Leciejewski et al. [38] compared MC-LR degradation by UV222 and UV254, highlighting the importance of wavelength selection for optimizing degradation efficiency. Despite these advances, no study has specifically investigated whether UV-generated MC-LR photoisomers can serve as substrates for subsequent bacterial degradation, nor has the enzymatic mechanism underlying such a sequential process been elucidated at the molecular level.

However, critical knowledge gaps persist, such as insufficient evidence for the degradation efficiency and the unknown molecular mechanisms. Unlike previous studies that focused on UV-based advanced oxidation processes (AOPs) for MC-LR degradation [18,26,28], or standalone biological treatment [20,22], this study specifically demonstrates that UV-C-induced photoisomerization enhances subsequent enzymatic biodegradation of MC-LR by *Sphingopyxis* sp. m6 and provides enzyme-level evidence for parallel degradation pathways originating from both the parent toxin and its photoisomers. This work provides a mechanistic understanding of the degradation pathway and proposes a practical technological strategy for efficient MC-LR degradation in water.

## 2. Results

### 2.1. Evaluation of UV Degradation Efficiency and Influence of Environmental Factors

**Figure 1:**
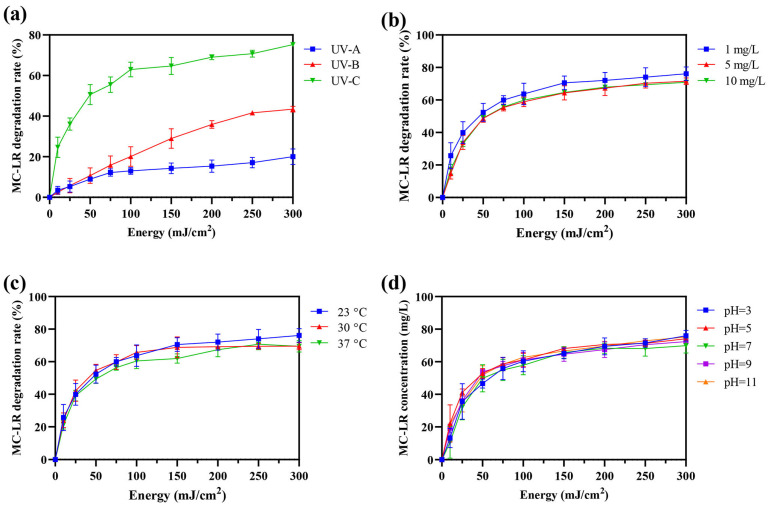
UV degradation efficiency of MC-LR and influencing factors. (**a**) Comparison of MC-LR degradation efficiency under UV-A (365 nm), UV-B (302 nm), and UV-C (254 nm) irradiation at doses of 50 and 100 mJ/cm^2^. (**b**) Effect of initial MC-LR concentration (1, 5, and 10 mg/L) on UV-C degradation efficiency at 50 mJ/cm^2^. (**c**) Effect of temperature (20, 30, and 37 °C) on UV-C degradation efficiency. (**d**) Effect of pH (3–11) on UV-C degradation efficiency. Data are presented as mean ± SD (*n* = 3).

While environmental concentrations typically range from 1 to 100 μg/L, with peak levels occasionally exceeding 1 mg/L in severely affected water bodies during bloom lysis events, laboratory studies often employ elevated concentrations (0.1–10 mg/L) to facilitate mechanistic investigation, product identification, and kinetic analysis [9,10,11,22,26,36]. In this study, the degradation efficiencies of MC-LR (1 mg/L) under irradiation by UV-A, UV-B, and UV-C were evaluated. As shown in Figure 1a, UV-C demonstrated significantly superior degradation efficacy, achieving 50.66% and 62.95% removal at 50 mJ/cm^2^ and 100 mJ/cm^2^, respectively. In contrast, UV-A and UV-B showed markedly lower efficiencies (8.93% and 13.01% at 50 mJ/cm^2^; 10.72% and 20.07% at 100 mJ/cm^2^). Based on its highest performance, UV-C was selected for all subsequent studies. For UV-C treatment, the energy-normalized degradation efficiency was 1.01% per mJ/cm^2^ at 50 mJ/cm^2^, compared to 0.63% per mJ/cm^2^ at 100 mJ/cm^2^, confirming the higher energy efficiency of the lower dose. Therefore, 50 mJ/cm^2^ UV-C treatment was chosen as the standard irradiation condition.

The effects of key environmental factors on UV-C degradation efficiency were further investigated (Figure 1b–d). Varying the initial MC-LR concentration (1, 5, and 10 mg/L) resulted in only marginal, statistically non-significant differences in degradation rate (Figure 1b). Similarly, no significant influence was observed across a temperature range of 20–37 °C (Figure 1c) or a pH range of 3–11 (Figure 1d). No degradation was detected in the non-irradiated control. These results indicate that, within the tested ranges, the efficiency of UV-C-mediated MC-LR degradation in pure water is not substantially affected by initial concentration, temperature, or pH.

### 2.2. MC-LR Degradation by Sequential UV-C and Sphingopyxis sp. m6

**Figure 2:**
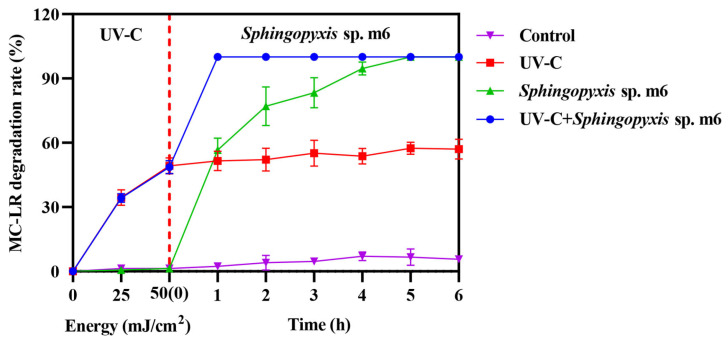
Degradation of MC-LR by individual and sequential treatments. Data are presented as mean ± SD (*n* = 3).

The MC-LR (1 mg/L) degradation ability of combining UV-C irradiation (50 mJ/cm^2^) with biodegradation by *Sphingopyxis* sp. m6 was evaluated by comparing the across single and combined treatments (Figure 2). While both the *Sphingopyxis* sp. m6 treatment alone and the sequential UV-C/m6 treatment achieved high degradation efficiencies (100%) within 5 h, their kinetic profiles differed substantially.

The UV-C pretreatment alone rapidly degraded approximately 50% of MC-LR within the initial irradiation phase (0 h time point, representing immediate post-irradiation sampling). Subsequently, the MC-LR concentration in this group remained relatively stable, plateauing at around 57% degradation after 5 h, indicating the limitation of UV-C in achieving complete mineralization. In contrast, the *Sphingopyxis* sp. m6-only treatment exhibited a progressive degradation, reaching complete degradation after 5 h.

The sequential combination demonstrated a pronounced synergistic effect. Following the initial UV-C pretreatment (50% degradation), the subsequent introduction of *Sphingopyxis* sp. m6 led to the rapid and complete removal of the remaining MC-LR within just 1 h of biological treatment. This represents a significant acceleration compared to the standalone biological process. No appreciable degradation was observed in the untreated control. These results clearly indicate that the sequential UV-C and *Sphingopyxis* sp. m6 treatment system not only enhanced the overall degradation efficiency but also drastically reduced the time required to achieve complete MC-LR removal compared to either individual method.

### 2.3. UV-C Pretreatment Induces MC-LR Photoisomerization

**Figure 3:**
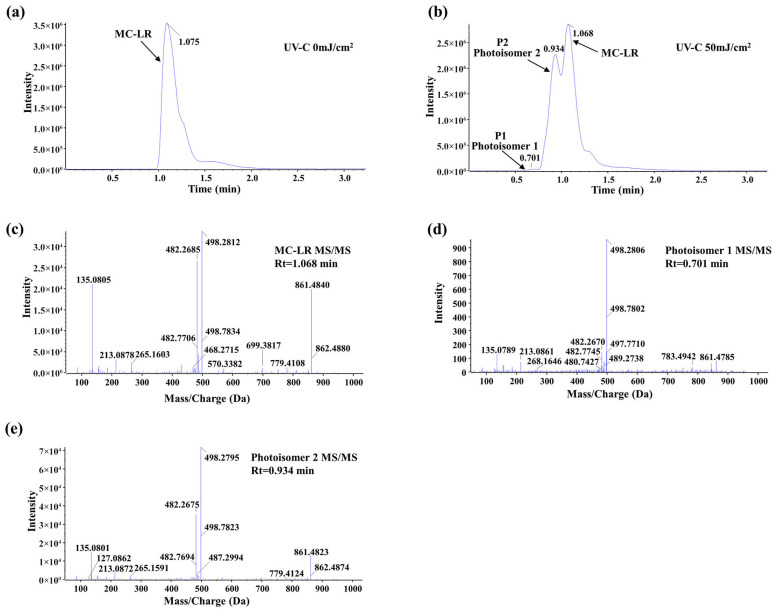
UPLC-MS/MS analysis of UV-C-induced MC-LR photoisomerization. (**a**) Chromatogram of parent MC-LR before UV irradiation (Rt = 1.075 min). (**b**) Chromatogram after UV-C irradiation (50 mJ/cm^2^), showing the appearance of two new peaks: Photoisomer 1 (P1, Rt = 0.701 min) and Photoisomer 2 (P2, Rt = 0.934 min). (**c**) MS/MS spectrum of parent MC-LR. (**d**) MS/MS spectrum of Photoisomer 1. (**e**) MS/MS spectrum of Photoisomer 2.

The molecular transformation of MC-LR upon UV-C irradiation (50 mJ/cm^2^) was investigated by UPLC-MS/MS analysis. As shown in the chromatograms (Figure 3a,b), the peak corresponding to the parent MC-LR (retention time, Rt = 1.068 min) decreased significantly after irradiation. Concurrently, two new distinct chromatographic peaks emerged at Rt = 0.701 min and Rt = 0.934 min, designated as P1 and P2, respectively. Notably, the peak intensity of P2 was substantially higher than that of P1 under the applied conditions.

To characterize these products, their MS/MS fragmentation patterns were analyzed and compared with that of the parent MC-LR (Figure 3c–e). All three compounds shared key diagnostic fragment ions, including *m*/*z* 135.0805 (PhCH_2_CHOMe^+^), 213.0878 (Glu-Mdha+H^+^), and 861.4840 (MC-LR-PhCh_2_CHOMe), confirming that the new products retain the core molecular skeleton of MC-LR. However, the relative abundances of these and other fragment ions differed discernibly among the parent compound and the two new peaks. This combination of identical molecular mass (implied by the same precursor ion), similar but distinct fragmentation profiles, and altered chromatographic behavior provides strong evidence that P1 and P2 are consistent with MC-LR photoisomers formed via UV-C-induced structural rearrangement, rather than products of oxidative cleavage or other decompositions. Referring to the stereochemistry of photoisomers characterized by nuclear magnetic resonance in the previous literature, the photoisomers in this study are likely two of 4(Z)-Adda MC-LR, 6(Z)-Adda MC-LR, and 4(Z),6(Z)-Adda MC-LR [32,36].

### 2.4. Identification of Intermediate Products

A comprehensive analysis of the products generated during the degradation of UV-C-pretreated MC-LR by *Sphingopyxis* sp. m6 was conducted using UPLC-MS/MS. A total of six distinct degradation intermediates were identified (Table 1). A key finding was the detection of two parallel series of degradation products: one corresponding to the established pathway from parent MC-LR (designated as series “1”, including Linearized MC-LR 1, Tetrapeptide 1, and Adda 1), and a second, novel series (designated as series “2”, including Linearized MC-LR 2, Tetrapeptide 2, and Adda 2) characterized by different chromatographic elution times and distinct MS/MS fragmentation patterns, despite sharing identical molecular ions with their series “1” counterparts (Figures S1–S3). Notably, the series “2” products are proposed to originate specifically from the bacterial degradation of MC-LR Photoisomer 2. The degradation products of MC-LR Photoisomer 1 were not detected in this study, likely due to its relatively low concentration following UV-C pretreatment falling below the instrument’s detection limit. Finally, all detected products were degraded by *Sphingopyxis* sp. m6.

### 2.5. Elucidation of the Degradation Pathway Using Recombinant Mlr Enzymes

To delineate the enzymatic steps involved in the biodegradation process, both parent MC-LR and UV-C-pretreated MC-LR were degraded by heterologously expressed MlrA, MlrB, and MlrC enzymes in vitro. The degradation of parent MC-LR by the recombinant enzymes proceeded via the established pathway: MlrA catalyzed the hydrolytic cleavage of the Adda-Arg bond, yielding Linearized MC-LR 1. This intermediate was subsequently processed by MlrB to Tetrapeptide 1, and finally, MlrC acted on both Linearized MC-LR 1 and Tetrapeptide 1 to release Adda 1. These products are consistent with those identified from whole-cell *Sphingopyxis* sp. m6 degradation (Table 1). Crucially, analysis of the UV-C-pretreated MC-LR mixture revealed that the recombinant Mlr enzymes can also degrade MC-LR photoisomers rapidly. Alongside the products from the parent MC-LR pathway, the second parallel series of intermediates was detected (Linearized MC-LR 2, Tetrapeptide 2, and Adda 2, Table 1). This confirms that MlrA can hydrolyze the isomeric MC-LR structures, MlrB can further cleave the resulting linearized isomers, and MlrC retains activity toward these alternative substrates to release the modified Adda moiety.

**Figure 4:**
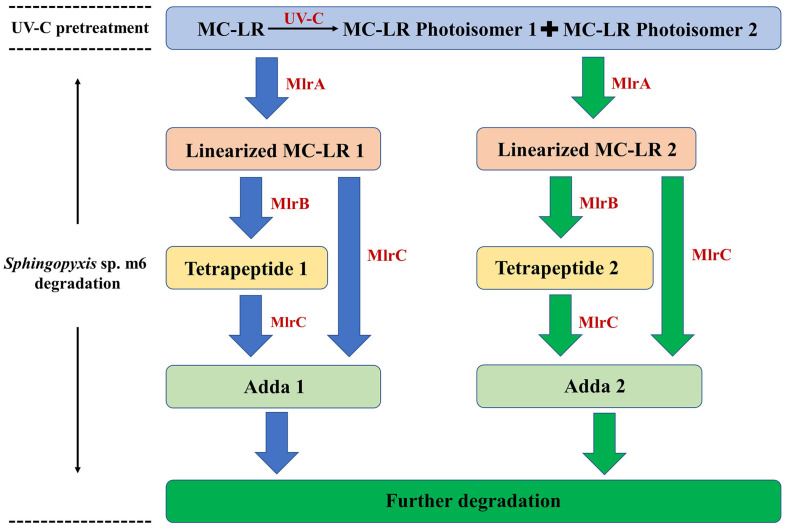
Proposed degradation pathway of MC-LR in the sequential UV-C and *Sphingopyxis* sp. m6 treatment system. The schematic illustrates the dual pathways: the hydrolysis of parent MC-LR (blue arrows) and the concurrent hydrolysis of UV-C-induced MC-LR photoisomers (green arrows), catalyzed by the specific Mlr enzymes.

Based on these enzymatic product profiles, a comprehensive dual-pathway degradation pathway for the UV-C irradiation and *Sphingopyxis* sp. m6 biodegradation coupling system is proposed (Figure 4). The process initiates with UV-C irradiation, which photoisomerizes parent MC-LR primarily into MC-LR Photoisomer 2 (and a minor amount of Photoisomer 1). Subsequently, MlrA enzyme hydrolyzes both the residual parent MC-LR and the photoisomers, leading to the formation of Linearized MC-LR 1 and Linearized MC-LR 2, respectively. These linearized products are then independently processed by MlrB into Tetrapeptide 1 and Tetrapeptide 2. Finally, MlrC cleaves these tetrapeptides (and potentially the linearized precursors) to release Adda 1 and Adda 2, which are ultimately mineralized. In summary, the Mlr enzymes of *Sphingopyxis* sp. m6 can process UV-C-generated MC-LR photoisomers, thereby providing a direct molecular understanding for the observed synergistic degradation.

### 2.6. MC-LR Detoxification by Sequential UV-C and Sphingopyxis sp. m6 Treatment

**Figure 5:**
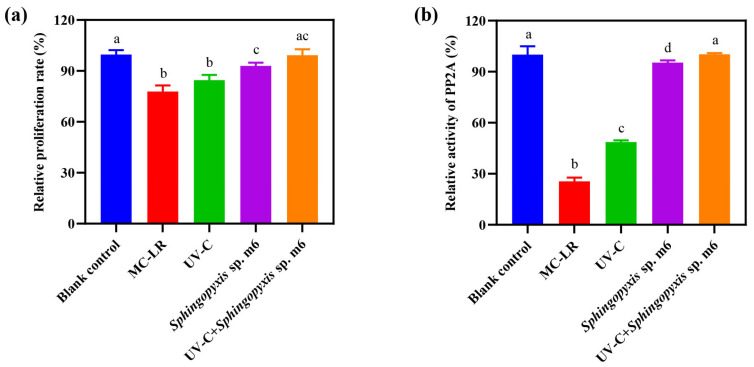
Toxicity assessment of MC-LR and its degradation products. (**a**) Relative cell proliferation rates determined by CCK-8 assay after 72 h exposure to reaction mixtures from different treatment groups. (**b**) Relative protein phosphatase 2A (PP2A) activity in HepG2 cell lysates. Data are presented as mean ± SD (*n* = 6). Different lowercase letters above bars indicate statistically significant differences (*p* ≤ 0.05) between treatment groups.

The detoxification efficacy of the sequential UV-C and *Sphingopyxis* sp. m6 treatment on MC-LR was evaluated using HepG2 cells, with cytotoxicity and protein phosphatase 2A (PP2A) inhibitory activity as endpoints (Figure 5). As Figure 5a shows, MC-LR significantly reduced the relative cell proliferation rate to 77.83% compared to the blank control (100%). While UV-C pretreatment alone slightly mitigated this cytotoxicity (84.53%), the difference from the MC-LR group was not statistically significant. In contrast, both the *Sphingopyxis* sp. m6 treatment alone and the sequential UV-C/m6 treatment effectively restored cell proliferation. No significant difference was observed between the sequential treatment group (99.22%) and the blank control. The detoxification efficacy based on cytotoxicity was ranked as UV-C+*Sphingopyxis* sp. m6 = *Sphingopyxis* sp. m6 > UV = MC-LR.

The inhibition of PP2A activity, a specific mechanism of MC-LR toxicity, was also evaluated (Figure 5b). MC-LR exposure strongly inhibited PP2A activity (relative activity: 25.56% vs. 100% in the blank control). UV-C pretreatment alone provided a modest but significant recovery (48.67%). The most effective restoration was achieved by biological treatments. The activity in the *Sphingopyxis* sp. m6 alone group was 95.32%, and it reached 100.23% in the sequential UV-C/m6 group, which was statistically equivalent to that of the blank control. The detoxification efficacy based on PP2A activity was ranked as UV-C+*Sphingopyxis* sp. m6 > *Sphingopyxis* sp. m6 > UV > MC-LR. In summary, the sequential UV-C and *Sphingopyxis* sp. m6 treatment not only achieved rapid and complete MC-LR degradation but also effectively eliminated both the general cytotoxicity and the specific enzymatic toxicity associated with MC-LR and its degradation intermediates.

### 2.7. Condition Optimization of MC-LR Degradation by Coupling UV-C Irradiation and Sphingopyxis sp. m6 Biodegradation

The degradation efficiency of MC-LR by the sequential UV-C and *Sphingopyxis* sp. m6 treatment is influenced by various environmental factors, which may exhibit interactive effects. Response surface methodology (RSM), a statistical design for bioprocess optimization, was employed to optimize these interdependent experimental parameters and determine the optimal conditions for MC-LR degradation.

**Figure 6:**
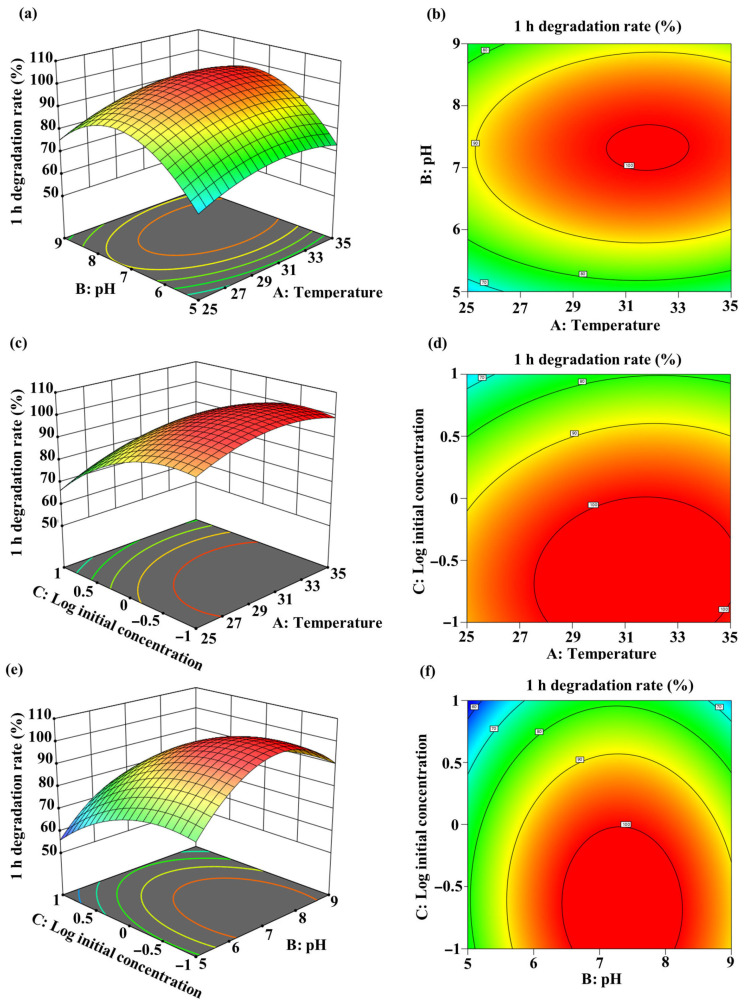
Response surface methodology optimization of MC-LR degradation by the sequential UV-C and *Sphingopyxis* sp. m6 treatment system. Three-dimensional response surface plots (**a**,**c**,**e**) and two-dimensional contour plots (**b**,**d**,**f**) showing the interactive effects of temperature, pH, and initial MC-LR concentration on the 1 h degradation efficiency. (**a**,**b**) Interaction between temperature and pH at fixed initial concentration (1 mg/L). (**c**,**d**) Interaction between temperature and log_10_(initial concentration) at a fixed pH of 7. (**e**,**f**) Interaction between pH and log_10_(initial concentration) at fixed temperature (30 °C).

A quadratic model was developed with the best fit to the experimental data, represented by the equation Y = −372.43187 + 15.1968A + 63.12325B − 16.5625C + 0.07975AB + 0.268AC − 0.47625BC − 0.24788A^2^ − 4.48237B^2^ − 9.097C^2^ (where Y = 1 h degradation rate (%), A = temperature (°C), B = pH, C = log_10_(initial MC-LR concentration (mg/L)). Analysis of variance (ANOVA) for the model parameters is presented in Table S1. The linear and quadratic coefficients for temperature (A), pH (B), and log_10_(initial MC-LR concentration) (C) significantly influenced MC-LR biodegradation (*p* < 0.05). Three-dimensional (3D) response surface plots and two-dimensional (2D) contour plots illustrate the interactive effects of temperature, pH, and log_10_(initial MC-LR concentration) on degradation efficiency (Figure 6). The optimal degradation conditions were determined as: temperature: 31.49 °C; pH: 7.36; and log_10_(initial MC-LR concentration): −0.64 (equivalent to 0.23 mg/L). Under these conditions, the predicted maximum degradation efficiency reached 100%.

## 3. Discussion

This study elucidates a novel and efficient strategy for MC-LR degradation based on the coupling of UV-C irradiation and *Sphingopyxis* sp. m6 biodegradation. The core finding is that pretreatment with a low UV-C dose (50 mJ/cm^2^) significantly enhances the rate and efficiency of subsequent biodegradation by inducing the MC-LR photoisomerization.

### 3.1. MC-LR Isomerization by UV-C

UV-C (254 nm) exhibits significantly higher conversion efficiency for MC-LR compared to UV-A (365 nm) and UV-B (302 nm) in the absence of exogenous photosensitizers (Figure 1a). This is primarily attributed to the close match between the energy of 254 nm photons and the electronic transition energy of the key chromophore in MC-LR (the conjugated diene in the Adda with λmax of 238 nm) leading to degradation predominantly via the direct photolysis pathway [33,36,37]. While prior studies noted similar degradation efficiencies for wavelengths of 238, 242, and 254 nm and higher rates for UV-C 222 nm, a critical factor is the irradiation dose, which dictates the reaction pathway and product profile [26,36,38]. The 50 mJ/cm^2^ dose employed here can efficiently induce cis–trans-geometric isomerization at the C4-C5 and C6-C7 double bonds of the Adda (generating Photoisomers 1 and 2), while largely avoiding irreversible photolysis or decarboxylation side reactions associated with higher doses [26,36,37]. This selective photochemical transformation converts MC-LR from a “recalcitrant” parent molecule into photoisomers that serve as “readily treatable” substrates for subsequent biodegradation. Our experiments confirmed that this low-dose pretreatment strategy is minimally affected by initial concentration, temperature, or pH (Figure 1b–d), demonstrating its technical robustness for potential application in complex aquatic environments.

### 3.2. Dual-Pathway Degradation by Sphingopyxis sp. m6

The analysis of UV-C pretreatment products revealed the starting point of the biodegradation by *Sphingopyxis* sp. m6 (Figure 3). UPLC-MS/MS data indicate that UV-C irradiation primarily generates two MC-LR photoisomers (P1 and P2). By tracking subsequent biodegradation products, this study indicates a parallel dual-pathway degradation model (Figure 4). In addition to the classical pathway from the parent MC-LR (yielding Linearized MC-LR 1, Tetrapeptide 1, and Adda 1), we clearly identified a distinct degradation pathway originating from the photoisomers (primarily P2), generating Linearized MC-LR 2, Tetrapeptide 2, and Adda 2. This suggests that *Sphingopyxis* sp. m6 can process both the parent compound and the UV-pretreated isomeric substrates in parallel, thereby accelerating the biomineralization process of pollutants at the molecular level.

### 3.3. Substrate Promiscuity of the Mlr Enzyme

The degradation experiments by recombinant enzymes provided direct evidence that the Mlr enzyme can degrade MC-LR photoisomers. This explains the acceleration of the overall biodegradation rate in the UV-C+*Sphingopyxis* sp. m6 group (Figure 2). We hypothesize that the conformational bending of the Adda chain, caused by geometric isomerization, may render the Adda-Arg peptide bond more accessible to MlrA. However, this mechanistic interpretation remains to be experimentally validated. Furthermore, the presence of auxiliary enzyme systems beyond the canonical Mlr pathway in *Sphingopyxis* sp. m6 cannot be ruled out. Such potential enzymes (e.g., non-specific peptidases, oxidoreductases) may cooperate in downstream mineralization steps, and the photoisomers might more efficiently induce the expression of these auxiliary systems, thereby accelerating the overall metabolic flux.

### 3.4. Efficient Detoxification and Application Potential

Cytotoxicity assays confirmed that while UV-C treatment alone (50 mJ/cm^2^) reduced MC-LR concentration by approximately 50%, its products showed limited recovery in cell proliferation and PP2A activity inhibition, indicating residual biotoxicity. In contrast, treatment with *Sphingopyxis* sp. m6 alone was effective in detoxification but required a longer duration (5 h). Remarkably, the UV-C/*Sphingopyxis* sp. m6 sequential treatment not only achieved complete degradation within 1 h but also restored the toxicity endpoints (cell proliferation and PP2A inhibition) to levels statistically indistinguishable from the blank control. While our results demonstrate effective detoxification of the mixture, the potential toxicity of individual intermediates was not evaluated. A recent study by Yu et al. [39] reported that some MC-LR biodegradation products retained partial PP2A inhibitory activity (2.8–43.5% of MC-LR), highlighting the importance of comprehensive toxicity assessment. In our study, the complete restoration of both cell proliferation and PP2A activity to control levels suggests that the sequential treatment effectively eliminates the primary toxic moieties. The enhanced MC-LR degradation and reduced biological toxicity were also observed in a combined biochar and electrolysis biofilters system, containing the electrolysis, adsorption and biodegradation processes [40]. This “physicochemicaactivation–biological mineralization” model may provide a promising strategy for emergency or advanced treatment to control seasonal algal bloom risks in source waters or contamination in drinking water distribution systems. However, validation under environmentally relevant MC concentrations and natural water matrices is still required

An ELISA-based assay targeting the Adda moiety is widely used for monitoring MC-like immunoreactivity in water [41]. Because UV-C pretreatment generates MC-LR geometric isomers and biodegradation yields Adda-related intermediates (Table 1), potential antibody cross-reactivity may occur, leading to discrepancies between immunoreactivity and functional toxicity endpoints (PP2A inhibition and HepG2 assays). The Adda-specific antibody developed by Zeck et al. [41] recognizes a common epitope in MCs and their fragments containing Adda, including geometric isomers. In addition, sample processing steps (UV exposure, biodegradation, lyophilization and reconstitution) may influence ELISA performance through matrix effects or epitope alteration. Therefore, ELISA measurements in treated samples should be interpreted cautiously and ideally complemented by functional toxicity assays, as performed in this study. Our combined approach using both cytotoxicity and PP2A activity provides a more comprehensive assessment of detoxification efficacy.

### 3.5. Limitations and Future Perspectives

While this study focuses on elucidating the mechanism of low-dose UV-C pretreatment promoting biodegradation via isomerization, limitations point to essential future research directions. First, doses higher than used here may generate different photolytic products, and systematically evaluating their biodegradability and potential toxicity is crucial. Second, although the degradation pathway of P2 has been clarified, the fate of the lower-concentration P1 and potential degradation routes for other trace photoproducts require more investigation. Finally, all experiments were conducted in pure water. Previous studies have demonstrated that natural water matrices, containing dissolved organic matter and suspended particles, can significantly influence UV transmittance and the efficiency of photochemical processes due to light attenuation and radical scavenging [33,37]. Therefore, the effects of complex matrices in real environmental waters (e.g., dissolved organic matter, suspended particles) on UV transmittance and microbial activity remain to be validated in real water or at pilot scale. In addition, the relatively high experimental MC-LR concentration (1 mg/L) exceeds typical environmental levels (µg/L range). While this concentration was chosen to facilitate mechanistic investigation and product identification—a common practice in degradation pathway studies [22,26,36]—concentration-dependent kinetics may influence treatment performance. The efficacy of this coupling system against the lower environmental concentrations of MCs (1–100 µg/L) need to be further determined.

Based on these points, future research should focus on: (1) Atomic-level mechanistic insights: Resolving the crystal structures of Mlr enzymes complexed with MC-LR photoisomers to reveal the structural basis for their broad substrate recognition at the atomic level. (2) Process integration and engineering: Developing integrated reactor systems combining UV modules with immobilized bacteria/enzyme preparations to evaluate stability and efficacy under realistic conditions, including the effects of turbidity on UV fluence delivery and long-term maintenance of bacterial activity in continuous-flow systems. (3) Ecological safety assessment: Systematically investigating the impact of this coupling process on the structure and function of ecosystem in receiving water bodies to ensure environmental friendliness.

## 4. Material and Methods

### 4.1. Bacteria and Reagents

*Sphingopyxis* sp. m6 (Genbank, accession number: MF535105), as a representative MC-degrading strain, was used in this study. *E. coli* BL21 (DE3) containing HMBP-pET-28a vector, HMBP-pET28a-mlrA, HMBP-pET28a-mlrB, and HMBP-pET28a-mlrC recombinant plasmids was adopted to overexpress of MlrA, MlrB, and MlrC enzymes from *Sphingopyxis* sp. m6.

Standard MC-LR (≥95%, ENZO, Farmingdale, NY, USA) was used as degradation substrate. Methanol (Tedia, Fairfield, OH, USA), acetonitrile (Merck, Darmstadt, Germany), trifluoroacetic acid (TFA, Macklin, Shanghai, China), and formic acid (Fisher Scientific, Shanghai, China) were purchased to analyze MC-LR and its products. Cell Counting Kit-8 (CCK-8, Baosai Biotechnology, Hangzhou, China) and Serine/Threonine Phosphatase Assay System (Promega, Madison, WI, USA) were used to assess the toxicity of native MC-LR and its products.

### 4.2. MC-LR Degradation by UV Irradiation and Influencing Factors

UV lamps (λ = 365 nm UV-A, 302 nm UV-B, and 254 nm UV-C, Philips, Eindhoven, The Netherlands) were positioned to achieve identical surface irradiance (0.27 mW/cm^2^) at the sample plane. Samples (200 µL) were collected at cumulative irradiation doses of 0, 10, 25, 50, 75, 100, 150, 200, 250, and 300 mJ/cm^2^. Following centrifugation, supernatants were analyzed by HPLC to determine residual MC-LR concentration and identify the optimal UV wavelength.

Using the optimal wavelength, the effects of environmental factors were evaluated: initial MC-LR concentration (1, 5, 10 mg/L; 30 °C, pH of 7), reaction temperature (20, 30, 37 °C; 1 mg/L MC-LR, pH of 7), and pH (3, 5, 7, 9, 11; 1 mg/L MC-LR, 30 °C). Control groups received no UV irradiation. All experiments were performed in triplicate.

### 4.3. Degradation of MC-LR by UV-C and Sphingopyxis sp. m6

The degradation efficacy of MC-LR was compared across four experimental groups: UV-C irradiation alone (UV-C), *Sphingopyxis* sp. m6 alone (*Sphingopyxis* sp. m6), sequential UV-C and *Sphingopyxis* sp. m6 treatment (UV-C+ *Sphingopyxis* sp. m6), and an untreated control group (Control). For treatments involving *Sphingopyxis* sp. m6, OD600 was adjusted to 1, while the UV-C irradiation dose was set at 50 mJ/cm^2^. Aqueous solutions of MC-LR (1 mg/L, pH of 7) were incubated at 30 °C. Samples were collected from each group at designated time intervals, and the residual MC-LR concentration was quantified by HPLC.

### 4.4. Enzymatic Pathway Verification Using Recombinant Mlr Enzymes

To validate the enzymatic degradation pathway of MC-LR and its isomers by Sphinopyxis sp. m6, heterologously expressed MlrA, MlrB, and MlrC enzymes were used [42,43]. In vitro parent MC-LR (1 mg/L) and UV-C (50 mJ/cm^2^) pretreated MC-LR was added in MSM as degradation substrates, respectively. Five reaction groups were established, including empty vector control, MlrA alone, MlrA+MlrB, MlrA+MlrC, and MlrA+MlrB+MlrC (each enzyme at 50 µg/mL). Reactions proceeded at 30 °C with shaking and samples were quenched with methanol at intervals. UPLC-MS/MS was used to detect substrate depletion and product formation, elucidating the specific role of each enzyme in processing both native and isomeric MC-LR.

### 4.5. Analysis of MC-LR Concentration and Degradation Products

Samples were centrifuged (12,000× *g*, 15 min, 4 °C) and supernatants were transferred to vials. HPLC was performed to detect MC-LR concentration using a Zorbax Extend C18 column (4.6 × 150 mm, 5 µm; Agilent, Santa Clara, CA, USA). The detection wavelength was 238 nm and the injection volume were 20 µL. The mobile phase consisted of 0.05% (*v*/*v*) aqueous trifluoroacetic acid and methanol (47:53, *v*/*v*) delivered at a flow rate of 1.0 mL/min. UPLC-MS/MS equipped with an ACQUITY BEH C18 (1.7 μm, 2.1 × 50 mm, waters, Millford, MA, USA) was used to analyze the degradation products of MC-LR. The mobile phases were 0.1% formic acid aqueous solution and acetonitrile at a flow rate of 0.2 mL/min. The specific parameters referred to our previously published articles [44].

### 4.6. Detoxification Efficacy Assessment

Four treatment groups were prepared, including UV-C irradiation only (50 mJ/cm^2^; designated UV-C), *Sphingopyxis* sp. m6 only (6 h reaction; designated *Sphingopyxis* sp. m6), sequential UV-C irradiation (50 mJ/cm^2^) followed by immobilized microspheres (designated UV-C+ *Sphingopyxis* sp. m6), untreated MC-LR (designated MC-LR), and a toxin-free control (designated blank control; cell culture medium only). Reaction mixtures were lyophilized, redissolved in cell culture medium to 1/50 of the original volume, and sterilized by filtration as cell exposure media, ensuring that any residual toxicity would be amplified and detectable. HepG2 cells were seeded in 96-well plates and exposed to the above prepared media. The exposure media were replaced every 24 h for a total exposure duration of 72 h. Six replicate wells were used for each experimental group. Cytotoxicity was measured via CCK-8 assay, and PP2A activity was determined in cell lysates following kit protocols.

### 4.7. Optimization of MC-LR Degradation Conditions by RSM

RSM was used through Design-Expert software (Version 13.0) to analyze the main effects, interaction effects, and quadratic effects of the independent variables, modeling their relationship with the MC-LR degradation rate to predict the optimal degradation conditions. A Box–Behnken design (BBD) was implemented with three independent variables, each at three levels: temperature (A): 25 °C, 30 °C, 35 °C; pH (B): 5, 7, 9; initial MC-LR concentration (C): 0.1 mg/L, 1 mg/L, 10 mg/L (Table S2). The Box–Behnken design generated 17 experimental runs for detecting the optimal degradation conditions (Table S3).

### 4.8. Statistical Analysis

All experiments were performed in triplicate with three independent replicates. Data are expressed as the mean ± standard deviation (SD). MC-LR degradation rate (%) was calculated as [1 − (Ct/C_0_)] × 100, where Ct represents the MC-LR concentration at time t, and C_0_ represents the initial MC-LR concentration at time zero for each treatment group. Statistical analysis (one-way ANOVA, *p* ≤ 0.05) confirmed no significant differences in degradation rate across the tested ranges. For toxicity data, differences in toxicity endpoints between treatment groups were assessed (one-way ANOVA) and pairwise comparisons were conducted using Tukey’s honest significant difference (HSD) post hoc test. Statistical analysis was performed using SPSS software (Version 16.0) and *p* ≤ 0.05 was considered statistically significant.

## Figures and Tables

**Table 1:** UPLC-MS/MS identification of parent MC-LR and its degradation products.

Compound	Rt (min)	([M+2H]/2)^2+^/[M+H]^+^ (Da)	Main Fragments (Da)	Proposed Origin/Identity	MS/MS Spectrum
MC-LR	1.068	498.2812	482.2685, 861.4840, 135.0805	Parent MC-LR	Figure 3c
MC-LR Photoisomer 1	0.701	498.2806	135.0789, 213.0861, 861.4785	UV-C-induced isomer	Figure 3d
MC-LR Photoisomer 2	0.934	498.2795	482.2675, 135.0801, 861.4823	UV-C-induced isomer	Figure 3e
Linearized MC-LR 1	1.14	507.3014	135.0803, 879.4819, 408.7858	Hydrolysis of parent MC-LR	Figure S1b
Linearized MC-LR 2	0.912	507.2864	491.2726, 879.4964, 135.0782	Hydrolysis of a photoisomer	Figure S1c
Tetrapeptide 1	1.217	615.3401	583.2961, 597.3552, 481.2486	From Linearized MC-LR 1	Figure S2b
Tetrapeptide 2	1.021	615.3422	464.2353, 598.3154, 375.1848	From Linearized MC-LR 2	Figure S2c
Adda 1	1.211	332.2229	135.0799, 283.1694, 300.1989	From parent MC-LR pathway	Figure S3b
Adda 2	0.977	332.2243	300.2015, 256.2079, 135.0808	From photoisomer pathway	Figure S3c

## Data Availability

The original contributions presented in this study are included in the article/Supplementary Materials. Further inquiries can be directed to the corresponding authors.

## Supplementary Materials

The following supporting information can be downloaded at https://www.mdpi.com/article/10.3390/toxins18030136/s1. Figure S1: Chromatogram and MS/MS spectrum of Linearized MC-LR. (a) Extracted chromatogram of Linearized MC-LR. (b) MS/MS spectrum of Linearized MC-LR 1. (c) MS/MS spectrum of Linearized MC-LR 2; Figure S2: Chromatogram and MS/MS spectrum of Tetrapeptide. (a) Extracted chromatogram of Tetrapeptide. (b) MS/MS spectrum of Tetrapeptide 1. (c) MS/MS spectrum of Tetrapeptide 2; Figure S3: Chromatogram and MS/MS spectrum of Adda. (a) Extracted chromatogram of Adda. (b) MS/MS spectrum of Adda 1. (c) MS/MS spectrum of Adda 2; Table S1: Analysis of variance (ANOVA) for the fitted model parameters of MC-LR degradation by the sequential UV-C and *Sphingopyxis* sp. m6 system; Table S2: Factors influencing MC-LR degradation efficiency and their variation levels; Table S3: Box–Behnken Design scheme for detecting the optimal degradation conditions.
