# Supplementary material for: Sequential UV-C Irradiation and Sphingopyxis sp. m6 Biodegradation for Enhanced Degradation and Detoxification of Microcystin-LR
Source: Toxins (Basel). 2026 Mar 10;18(3):136. doi: 10.3390/toxins18030136 (PMC13030729; doi:10.3390/toxins18030136)
Supplement: Supplementary file 1 [file toxins-18-00136-s001.zip › toxins-4176489-supplementary.pdf]

Supplementary Materials: Sequential UV-C irradiation and *Sphingopyxis* sp. m6 biodegradation for enhanced degradation and detoxification of microcystin-LR

Qin Ding, Tongtong Liu, Zhuoxiao Li, Rongli Sun, Juan Zhang, Lihong Yin and Yuepu Pu

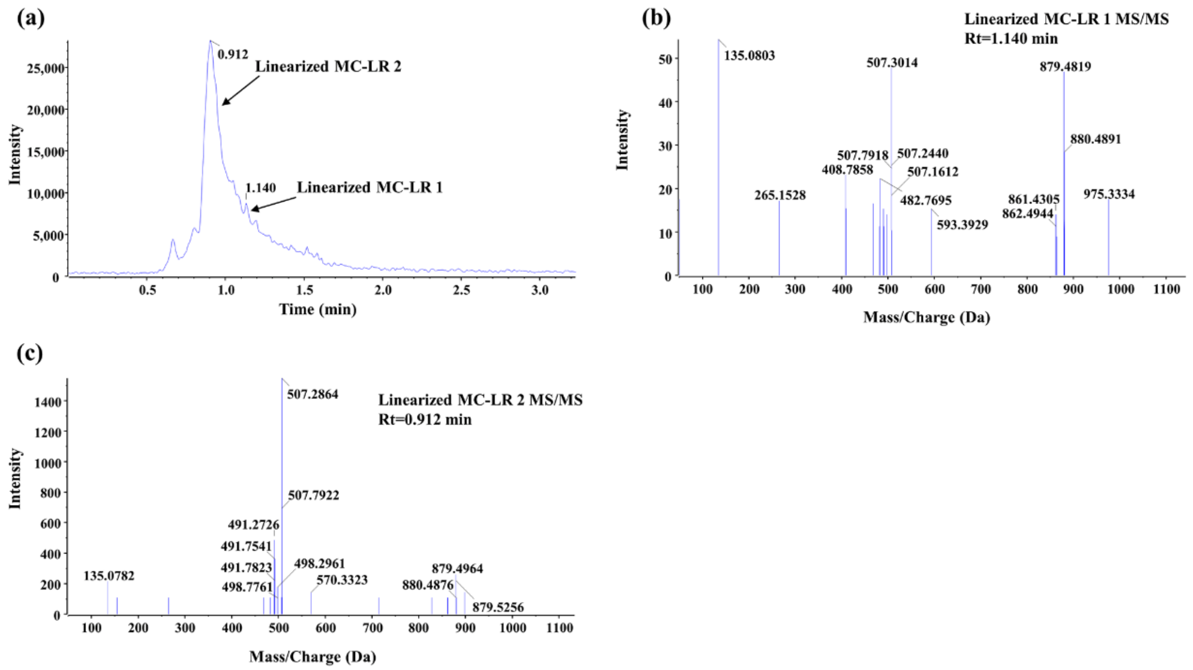

Figure S1. Chromatogram and MS/MS spectrum of Linearized MC-LR. (a) Extracted chromatogram of Linearized MC-LR. (b) MS/MS spectrum of Linearized MC-LR 1. (c) MS/MS spectrum of Linearized MC-LR 2.

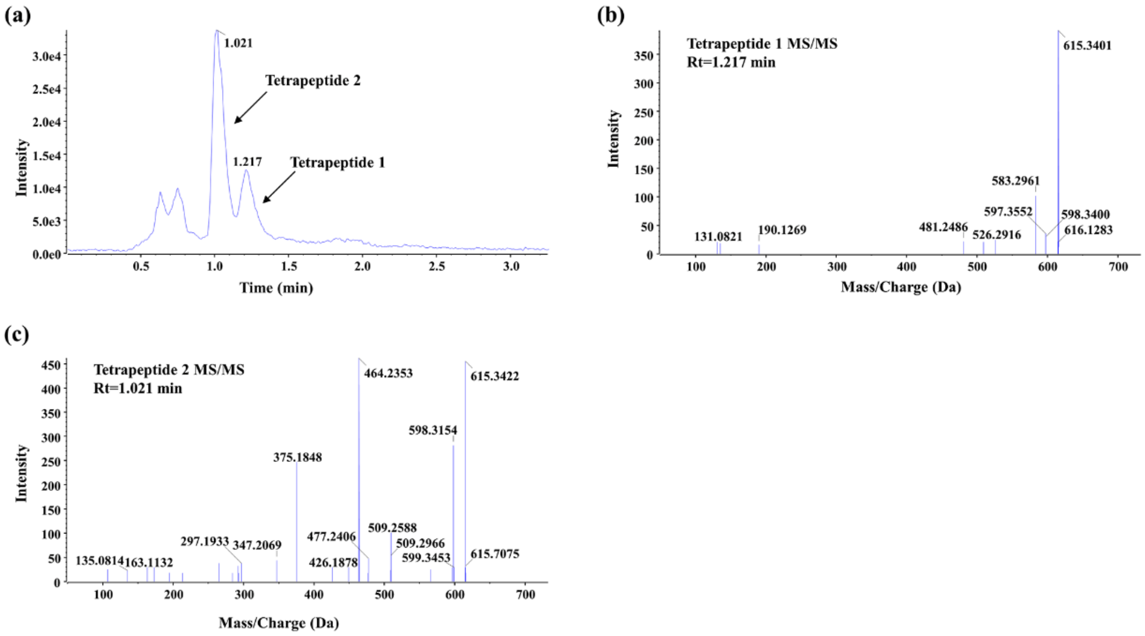

Figure S2. Chromatogram and MS/MS spectrum of Tetrapeptide. (a) Extracted chromatogram of Tetrapeptide. (b) MS/MS spectrum of Tetrapeptide 1. (c) MS/MS spectrum of Tetrapeptide 2.

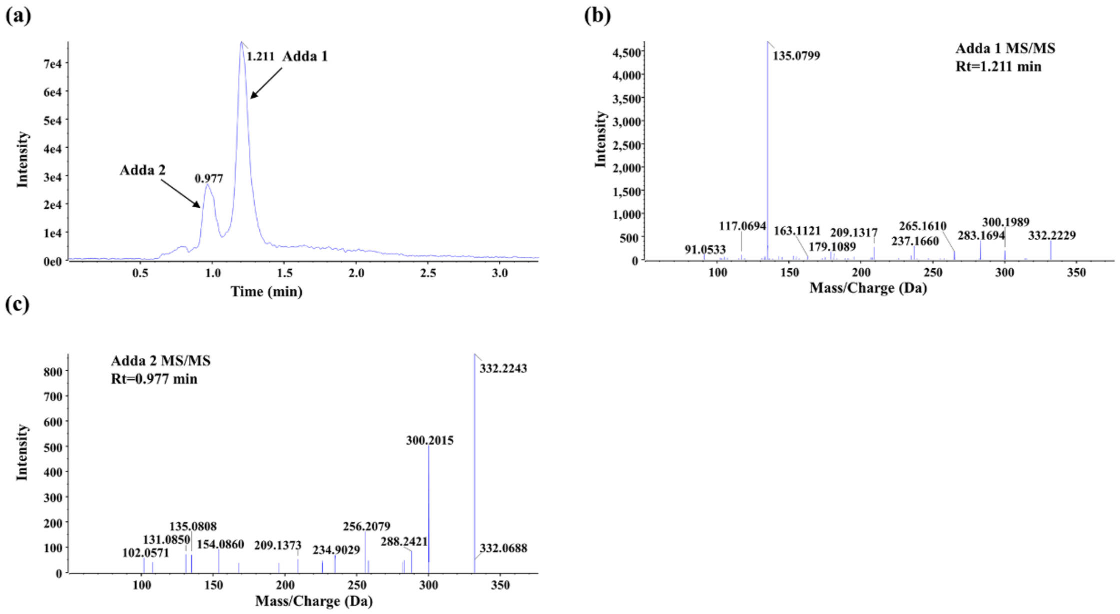

Figure S3. Chromatogram and MS/MS spectrum of Adda. (a) Extracted chromatogram of Adda. (b) MS/MS spectrum of Adda 1. (c) MS/MS spectrum of Adda 2.

Table S1. Analysis of variance (ANOVA) for the fitted model parameters of MC-LR degradation by the sequential UV-C and *Sphingopyxis* sp. m6 system.

Source	Sum of squares	Mean square	F-value	*P*-value
Model	3,568.02	396.45	46.77	< 0.0001
A-Temperature	155.67	155.67	18.36	0.0036
B-pH	244.21	244.21	28.81	0.001
C-Log initial concentration	1,124.57	1,124.57	132.66	< 0.0001
AB	2.54	2.54	0.3001	0.6008
AC	7.18	7.18	0.8473	0.3879
BC	3.63	3.63	0.4281	0.5338
A^2	161.7	161.7	19.08	0.0033
B^2	1353.55	1353.55	159.68	< 0.0001
C^2	348.44	348.44	41.11	0.0004
R^2 = 0.9836, Adjusted R^2 = 0.9626, Coefficient of variation = 3.48%。				

Table S2. Factors influencing MC-LR degradation efficiency and their variation levels.

Factor	Levels		
Temperature (A)	25	30	35
pH (B)	5	7	9
Log initial concentration (C)	-1	0	1

Table S3. Box-Behnken Design scheme for detecting the optimal degradation conditions.

Std	A: Temperature (°C)	B: pH	C: Log initial concentration (lgmg/L)	1 h degradation rate (%)
1	25	5	0	63.85
2	35	5	0	75.60
3	35	9	0	88.20
4	25	9	0	73.26
5	25	7	-1	96.55
6	35	7	-1	98.17
7	35	7	1	74.25
8	25	7	1	67.27
9	30	5	-1	76.24
10	30	9	-1	89.24
11	30	5	1	57.32
12	30	9	1	66.51
13	30	7	0	98.54
14	30	7	0	100.00
15	30	7	0	100.00
16	30	7	0	98.85
17	30	7	0	99.38
